# Supplementary material for: Automated lifespan determination across Caenorhabditis strains and species reveals assay-specific effects of chemical interventions
Source: GeroScience. 2019 Dec 10;41(6):945–60. doi: 10.1007/s11357-019-00108-9 (PMC6925072; doi:10.1007/s11357-019-00108-9)
Supplement: Supplementary file 1 — Summary of ALM lifespan data under baseline conditions, and comparison to median lifespan from comparable manual assays (PDF 159 kb) [file 11357_2019_108_MOESM1_ESM.pdf]

Online Resource 1 Summary of ALM lifespan data under baseline conditions, and comparison to median lifespan from comparable manual assays

		ALM								Manual	
Species	Strain	Number of deaths	Number censored	Total observed	Mean lifespan	SEM	Median lifespan	Lower 95% CI	Upper 95% CI	Median lifespan	% diff median LS from manual
C.elegans	N2	753	0	753	16.5	0.10	17.0	16.6	17.1	19	-11
	N2 PD1073	856	2	858	15.8	0.12	15.5	15.1	15.8	-	-
	CB4856	628	0	628	17.9	0.16	18.2	17.6	18.5	20	-9
	ED3040	698	3	701	15.2	0.12	14.8	14.5	15.0	20	-26
	JU1088	848	1	849	17.0	0.13	17.0	16.7	17.4	18	-5
	JU1652	670	3	673	17.8	0.18	17.8	17.5	18.4	21	-15
	JU775	675	3	678	17.2	0.17	16.6	16.1	17.1	21	-21
	MY16	660	5	665	16.5	0.14	16.8	16.3	17.3	18	-7
	QX1211	180	0	180	16.6	0.26	15.9	15.5	16.7	19	-16
C. briggsae	AF16	398	6	404	20.0	0.24	20.0	19.4	20.7	25	-20
	ED3092	575	1	576	22.5	0.20	23.1	22.8	23.4	25	-8
	HK104	578	4	582	27.9	0.26	29.1	28.7	29.6	33	-12
	JU1264	580	0	580	21.2	0.28	20.3	19.5	21.2	23	-12
	JU1348	524	0	524	19.2	0.25	18.5	17.9	19.0	23	-20
	JU726	377	0	377	18.2	0.24	18.0	17.5	18.5	19	-5
	NIC20	637	2	639	18.4	0.17	18.3	17.8	18.8	23	-20
	QR25	758	3	761	18.3	0.19	17.2	16.9	17.7	18	-4
C. tropicalis	JU1373	566	3	569	18.0	0.10	18.3	18.1	18.5	23	-21
	JU1630	334	1	335	19.8	0.19	20.0	19.6	20.5	26	-23
	NIC122	268	0	268	15.9	0.28	16.8	15.5	17.5	22	-23
	NIC58	544	0	544	15.7	0.21	15.3	14.6	16.3	21	-27
	QG131	572	0	572	18.5	0.17	18.6	18.3	19.0	22	-15
	QG834	779	3	782	18.8	0.12	19.0	18.7	19.1	23	-18
		Combined total		13,498							
