# Supplementary material for: Automated lifespan determination across Caenorhabditis strains and species reveals assay-specific effects of chemical interventions
Source: GeroScience. 2019 Dec 10;41(6):945–60. doi: 10.1007/s11357-019-00108-9 (PMC6925072; doi:10.1007/s11357-019-00108-9)
Supplement: Supplementary file 4 — Survival differences among C. elegans, C. briggsae and C. tropicalis species are not due to differences in censoring. (A) Survivorship curves for combined manual trials of each species indicated, when animals that died of unnatural causes were censored or (B) when all animals, including the censored population, were included in data analysis. (C) The basic shape and relative order of survival are maintained among species when data from all strains generated on the automated lifespan machines are combined. We did observe some strain-specific differences within species (see Online Resource 2) (PDF 334 kb) [file 11357_2019_108_MOESM4_ESM.pdf]

Online Resource 4 Survival differences among *C. elegans*, *C. briggsae* and *C. tropicalis* species are not due to differences in censoring

(A) Survivorship curves for combined manual trials of each species indicated, when animals that died of unnatural causes were censored or (B) when all animals, including the censored population, were included in data analysis. (C) The basic shape and relative order of survival are maintained among species when data from all strains generated on the automated lifespan machines are combined. We did observe some strain-specific differences within species (see Online Resource 2).

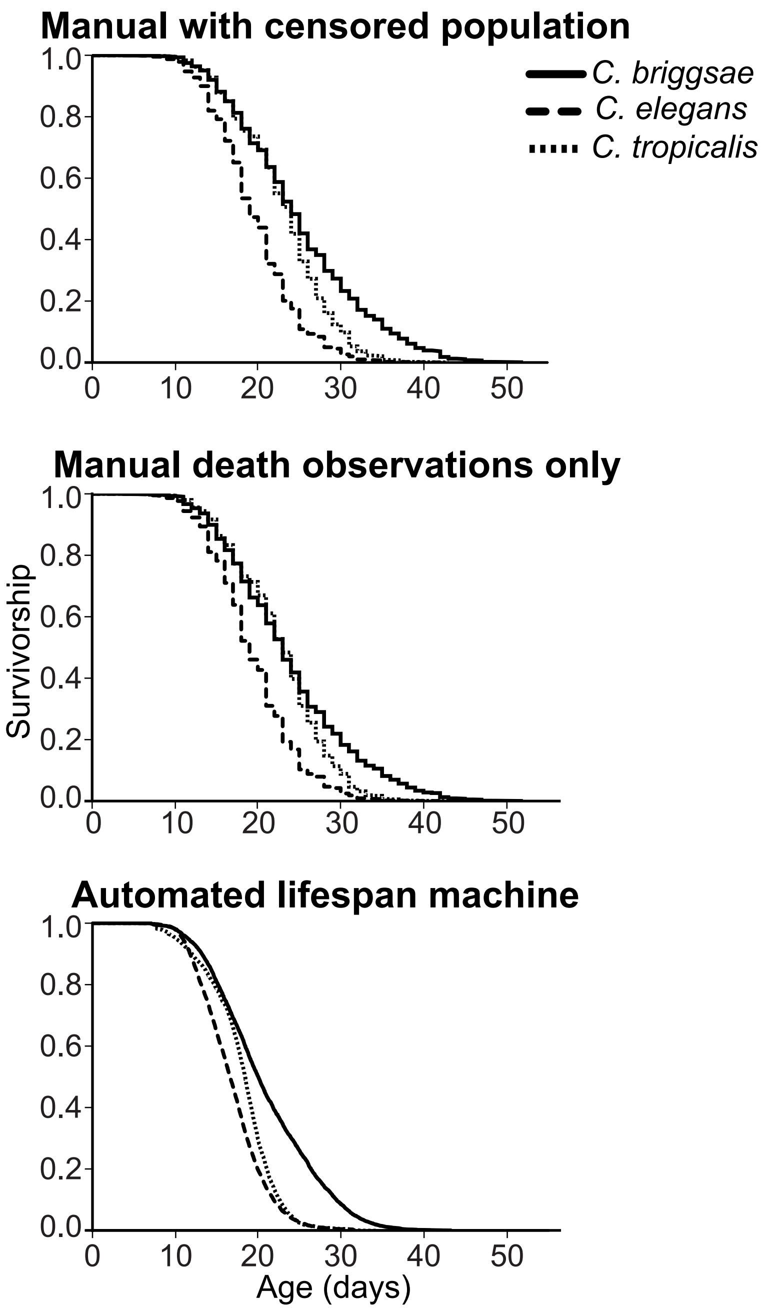
Manual with censored population
C. briggsae
C. elegans
C. tropicalis
Manual death observations only
Automated lifespan machine
Survivorship
Age (days)
1.0
0.8
0.6
0.4
0.2
0.0
0
10
20
30
40
50
