# Supplementary material for: Automated lifespan determination across Caenorhabditis strains and species reveals assay-specific effects of chemical interventions
Source: GeroScience. 2019 Dec 10;41(6):945–60. doi: 10.1007/s11357-019-00108-9 (PMC6925072; doi:10.1007/s11357-019-00108-9)
Supplement: Supplementary file 6 — Partitioning of variation for longevity across genetic background and experimental replicates within and among labs. Variance components were estimated as a randomized block design using a restricted maximum likelihood (REML) general linear model using the lme4 package of R. All factors were treated as random effects. Tests involving the fixed compound effects are reported in Online Resource 9. A. Variance component estimates for the natural log of individual lifespan from the 22-strain, three-species baseline longevity experiments. Sample sizes and summary statistics are presented in Online Resource 1 (PDF 132 kb) [file 11357_2019_108_MOESM6_ESM.pdf]

Online Resource 6 Partitioning of variation for longevity across genetic background and experimental replicates within and among labs. Variance components were estimated as a randomized block design using a restricted maximum likelihood (REML) general linear model using the *lme4* package of R. All factors were treated as random effects. Tests involving the fixed compound effects are reported in Online Resource 9.

A. Variance component estimates for the natural log of individual lifespan from the 22-strain, three-species baseline longevity experiments. Sample sizes and summary statistics are presented in Online Resource 1.

Source	Variance Component
Species	1.66E-03
Strain*Species	1.80E-03
Lab	3.08E-04
Lab*Species	9.26E-05
Lab*Strain	1.10E-03
Scanner*Lab	6.01E-09
Trial[Scanner, Lab]	3.98E-04
Plate_Trial[Trial, Scanner, Lab]	1.28E-03
Residual	9.29E-03
Total	1.59E-02

B. Average variance component estimates for the natural log of individual lifespan from the six-strain, three-compound experiments. See Online Resource 14 for a per-strain analysis of replication and samples sizes. Summary statistics are presented in Online Resource 11.

Source	Variance Component
Species	2.42E-03
Strain*Species	2.51E-03
Compound*Species	9.58E-04
Compound*Strain	3.75E-04
Lab	0.00E+00
Lab*Species	0.00E+00
Lab*Strain	1.55E-03
Compound*Lab	2.32E-05
Scanner*Lab	3.96E-10
Trial[Scanner, Lab]	5.34E-04
Plate_Trial[Trial, Scanner, Lab]	1.04E-03
Residual	9.52E-03
Total	1.89E-02

C. Average variance component estimates for the natural log of individual lifespan from the corresponding manual experiments. Sample sizes and summary statistics are presented in Online Resources 1 and 5.

Source	Variance Component	Lower 95% CI	Upper 95% CI	Percent of Total
Species	5.23E-02	0.00E+00	4.91E-01	32.5
Strain*Species	1.15E-02	3.08E-03	7.30E-02	7.1
Compound*Species	5.21E-03	1.79E-04	9.46E-03	3.2
Compound*Strain	3.26E-03	1.46E-03	7.78E-03	2.0
Lab	0.00E+00	0.00E+00	7.29E-03	0.0
Lab*Species	6.79E-04	0.00E+00	6.27E-03	0.4
Lab*Strain	3.94E-04	0.00E+00	2.06E-03	0.2
Compound*Lab	0.00E+00	0.00E+00	7.65E-04	0.0
Experimenter*Lab	2.34E-03	3.71E-04	7.37E-03	1.5
Trial[Experimenter, Lab]	3.46E-03	1.93E-03	6.06E-03	2.2
Plate_Trial[Trial, Experimenter, Lab]	8.29E-03	6.92E-03	9.90E-03	5.1
Residual	7.36E-02	7.19E-02	7.52E-02	45.7
Total	1.61E-01			100.0
