# Supplementary material for: Automated lifespan determination across Caenorhabditis strains and species reveals assay-specific effects of chemical interventions
Source: GeroScience. 2019 Dec 10;41(6):945–60. doi: 10.1007/s11357-019-00108-9 (PMC6925072; doi:10.1007/s11357-019-00108-9)
Supplement: Supplementary file 7 — Several lifespan compound interventions are reported similarly by manual and ALM analysis. The median lifespan under adult exposure to NP1 (a), propyl gallate (b) or resveratrol (c) are shown for three C. elegans (N2, JU775, and MY16) and C. briggsae (AF16, JU1348, and HK104) strains. Each point represents the median lifespan from an individual plate trial. The bars represent the mean +/− the standard error of the mean. Replicates were generated at the three CITP sites (Blue-Buck Institute, Green-Oregon and Red- Rutgers). Lifespans were measured for compound (circles) and vehicle control (triangles) conditions. Asterisks represent p values (****p<0.0001, *** p<0.001, ** p<0.01 and * p<0.05) from the CPH model when comparing the lifespans under compound exposure versus the lifespans exposed to the vehicle control (PDF 522 kb) [file 11357_2019_108_MOESM7_ESM.pdf]

Online Resource 7 Several lifespan compound interventions are reported similarly by manual and ALM analysis.

The median lifespan under adult exposure to NP1 (a), propyl gallate (b) or resveratrol (c) are shown for three *C. elegans* (N2, JU775, and MY16) and *C. briggsae* (AF16, JU1348, and HK104) strains. Each point represents the median lifespan from an individual plate trial. The bars represent the mean +/- the standard error of the mean. Replicates were generated at the three CITP sites (Blue-Buck Institute, Green-Oregon and Red- Rutgers). Lifespans were measured for compound (circles) and vehicle control (triangles) conditions. Asterisks represent p-values (****p<0.0001, *** p<0.001, ** p<0.01 and * p<0.05) from the CPH model when comparing the lifespans under compound exposure versus the lifespans exposed to the vehicle control.

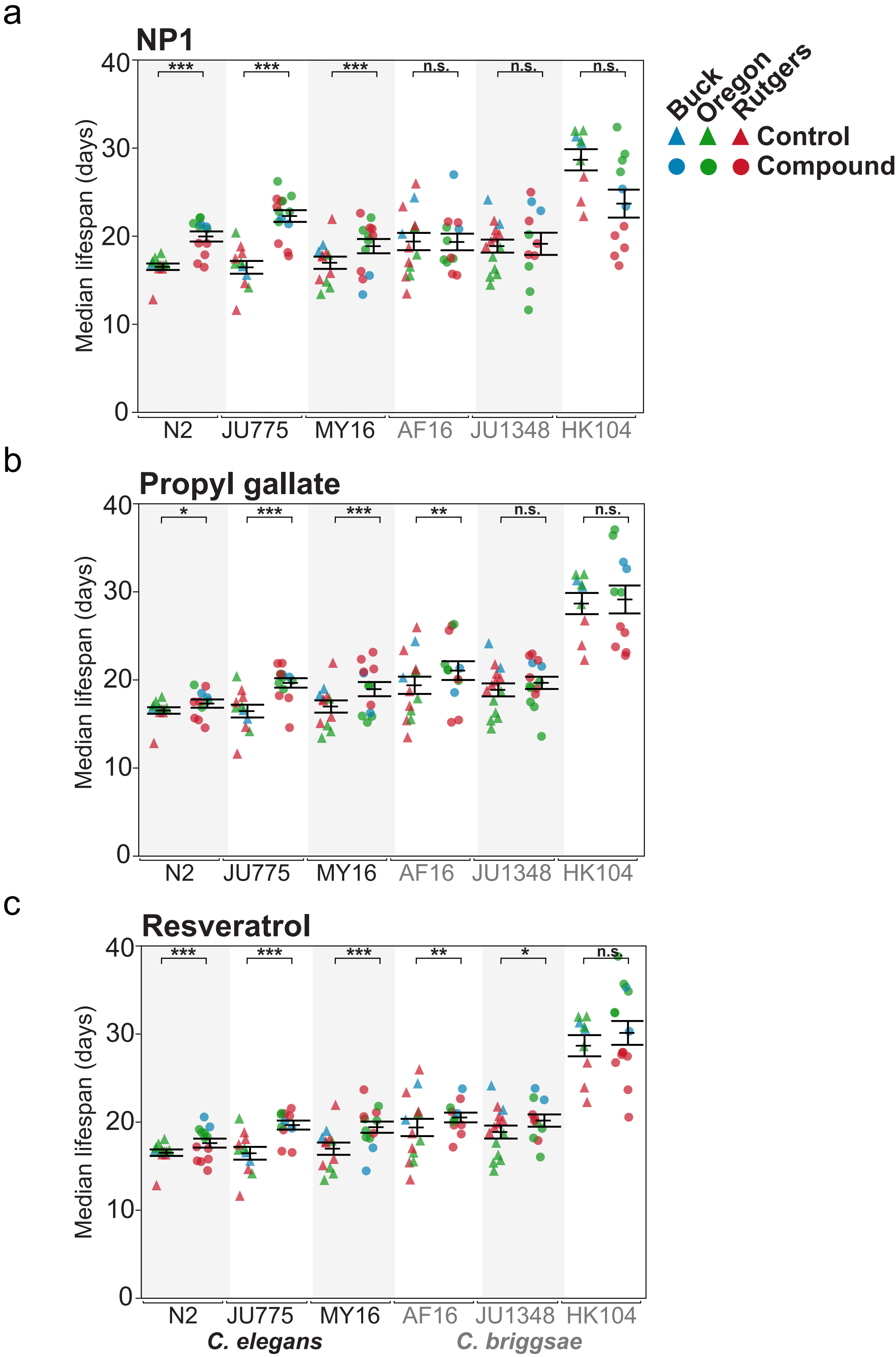

a
NP1
b
Propyl gallate
c
Resveratrol
Median lifespan (days)
0
10
20
30
40
***
n.s.
**
*
N2
JU775
MY16
AF16
JU1348
HK104
C. elegans
C. briggsae
Buck
Oregon
Rutgers
Control
Compound
