# Supplementary material for: Automated lifespan determination across Caenorhabditis strains and species reveals assay-specific effects of chemical interventions
Source: GeroScience. 2019 Dec 10;41(6):945–60. doi: 10.1007/s11357-019-00108-9 (PMC6925072; doi:10.1007/s11357-019-00108-9)
Supplement: Supplementary file 8 — Thioflavin T, but not α-ketoglutarate, lifespan effects are reversed by light exposure in ALM analysis. The median lifespans under adult exposure to thioflavin T (a) or α-ketoglutarate (b) are shown for three C. elegans (N2, JU775, and MY16) and C. briggsae (AF16, JU1348, and HK104) strains. Each point represents the median lifespan from an individual plate trial. The bars represent the mean +/- the standard error of the mean. Replicates were generated at three CITP sites (Blue-Buck Institute, Green-Oregon and Red- Rutgers). Lifespans were measured by standard automated survival analysis (vehicle control-triangles or compound - circles) or with automated lifespan analysis modified to accommodate light filtering (see materials and methods) (vehicle control-inverted triangles or compound-diamonds). Asterisks represent p values (****p<0.0001, *** p<0.001, ** p<0.01 and * p<0.05) from the CPH model for the shown comparisons (PDF 518 kb) [file 11357_2019_108_MOESM8_ESM.pdf]

Online Resource 8 Thioflavin T, but not α-ketoglutarate, lifespan effects are reversed by light exposure in ALM analysis.

The median lifespans under adult exposure to thioflavin T (a) or α-ketoglutarate (b) are shown for three *C. elegans* (N2, JU775, and MY16) and *C. briggsae* (AF16, JU1348, and HK104) strains. Each point represents the median lifespan from an individual plate trial. The bars represent the mean +/- the standard error of the mean. Replicates were generated at three CITP sites (Blue-Buck Institute, Green-Oregon and Red- Rutgers). Lifespans were measured by standard automated survival analysis (vehicle control-triangles or compound - circles) or with automated lifespan analysis modified to accommodate light filtering (see materials and methods) (vehicle control-inverted triangles or compound-diamonds). Asterisks represent *p*-values (****$p<0.0001$, *** $p<0.001$, ** $p<0.01$ and * $p<0.05$) from the CPH model for the shown comparisons.

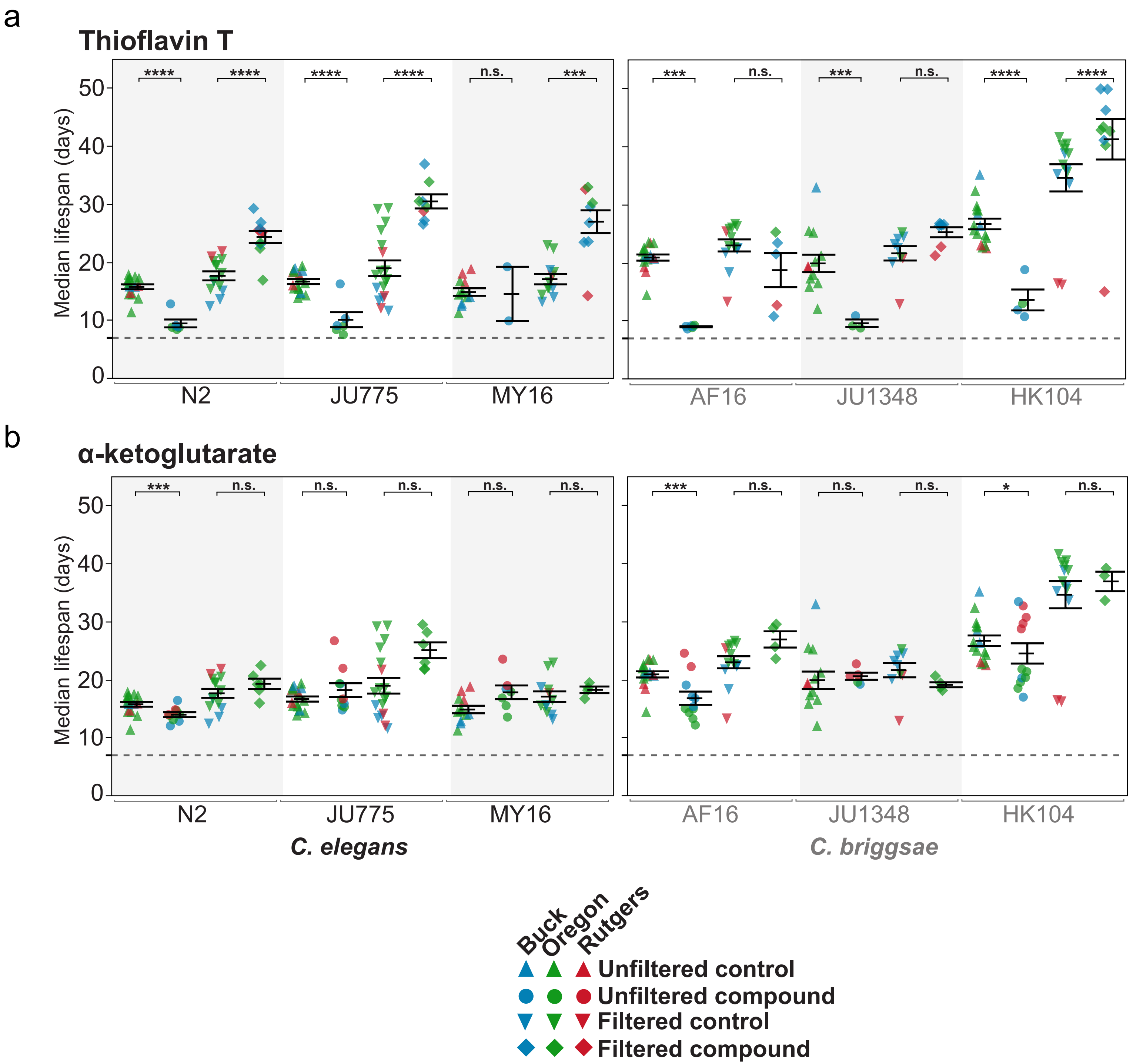
a
Thioflavin T
Median lifespan (days)
0
10
20
30
40
50
****
n.s.
***
N2
JU775
MY16
AF16
JU1348
HK104
b
α-ketoglutarate
*
C. elegans
C. briggsae
Buck
Oregon
Rutgers
Unfiltered control
Unfiltered compound
Filtered control
Filtered compound
