# Supplementary material for: Automated lifespan determination across Caenorhabditis strains and species reveals assay-specific effects of chemical interventions
Source: GeroScience. 2019 Dec 10;41(6):945–60. doi: 10.1007/s11357-019-00108-9 (PMC6925072; doi:10.1007/s11357-019-00108-9)
Supplement: Supplementary file 9 — Manual vs. ALM: approximate average return rates per plate (observed mean number of deaths versus expected number of animals at experiment start) for compound trials. The expected number of animals per plate for manual assays was 37.5, and for ALM assays was 50 (PDF 139 kb) [file 11357_2019_108_MOESM9_ESM.pdf]

Online Resource 9 Manual vs. ALM: approximate average return rates per plate (observed mean number of deaths versus expected number of animals at experiment start) for compound trials. The expected number of animals per plate for manual assays was 37.5, and for ALM assays was 50.

			Manual		ALM	
**Species**	**Strain**	**Compound**	**Average obs. deaths**	**% expected observed**	**Average obs. deaths**	**% expected observed**
C. elegans	N2	CTRL-H2O	33	88	32	64
		CTRL-DMSO	31	83	37	74
		AKG	30	80	36	72
		NP1	32	85	39	78
		Propyl gallate	29	77	37	74
		Resveratrol	30	80	37	74
		ThT	30	80	25	50
	JU775	CTRL-H2O	32	85	26	52
		CTRL-DMSO	29	77	35	70
		AKG	25	67	30	60
		NP1	29	77	35	70
		Propyl gallate	27	72	35	70
		Resveratrol	28	75	33	66
		ThT	27	72	19	38
	MY16	CTRL-H2O	30	80	26	52
		CTRL-DMSO	26	69	30	60
		AKG	25	67	26	52
		NP1	27	72	33	66
		Propyl gallate	24	64	29	58
		Resveratrol	22	59	30	60
		ThT	27	72	22	44
C. briggsae	AF16	CTRL-H2O	20	53	19	38
		CTRL-DMSO	21	56	21	42
		AKG	17	45	15	30
		NP1	18	48	23	46
		Propyl gallate	21	56	19	38
		Resveratrol	17	45	20	40
		ThT	19	51	20	40
	JU1348	CTRL-H2O	19	51	21	42
		CTRL-DMSO	20	53	23	46
		AKG	20	53	21	42
		NP1	23	61	25	50
		Propyl gallate	22	59	24	48
		Resveratrol	19	51	28	56
		ThT	18	48	24	48
	HK104	CTRL-H2O	28	75	31	62
		CTRL-DMSO	26	69	34	68
		AKG	24	64	27	54
		NP1	28	75	36	72
		Propyl gallate	25	67	33	66
		Resveratrol	27	72	33	66
		ThT	24	64	23	46
