# Supplementary material for: Automated lifespan determination across Caenorhabditis strains and species reveals assay-specific effects of chemical interventions
Source: GeroScience. 2019 Dec 10;41(6):945–60. doi: 10.1007/s11357-019-00108-9 (PMC6925072; doi:10.1007/s11357-019-00108-9)
Supplement: Supplementary file 10 — Summary of ALM lifespan data under compound treatment (NP1, propyl gallate, and resveratrol) conditions, and comparison to median lifespan from comparable manual assays (PDF 108 kb) [file 11357_2019_108_MOESM10_ESM.pdf]

Online Resource 10 Summary of ALM lifespan data under compound treatment (NP1, propyl gallate, and resveratrol) conditions, and comparison to median lifespan from comparable manual assays

			ALM								Manual	
**Species**	**Strain**	**Compound**	**Number of deaths**	**Number censored**	**Total observed**	**Mean lifespan**	**SEM**	**Median lifespan**	**Lower 95% CI**	**Upper 95% CI**	**Median lifespan**	**% diff med LS from manual**
C. elegans	JU775	CTRL-DMSO	382	0	382	16.8	0.23	16.6	15.8	17.4	17	-2
		NP1	492	0	492	21.6	0.20	22.3	21.7	22.7	21	6
		Propyl gallate	459	0	459	19.5	0.22	20.0	19.3	20.6	23	-13
		Resveratrol	362	0	362	19.9	0.27	19.8	18.8	20.5	24	-18
	MY16	CTRL-DMSO	359	1	360	17.2	0.18	17.3	16.6	17.7	15	16
		NP1	441	0	441	18.6	0.18	19.4	18.9	19.8	21	-8
		Propyl gallate	351	2	353	19.1	0.24	19.4	18.7	20.4	18	8
		Resveratrol	385	0	385	19.5	0.21	20.1	19.6	20.5	18	12
	N2	CTRL-DMSO	444	0	444	16.4	0.12	16.8	16.6	17.0	17	-1
		NP1	472	0	472	19.9	0.16	20.6	20.3	20.8	21	-2
		Propyl gallate	407	0	407	17.4	0.16	17.5	17.0	17.7	19	-8
		Resveratrol	483	0	483	17.6	0.15	17.7	17.3	18.1	19	-7
C. briggsae	AF16	CTRL-DMSO	299	0	299	19.4	0.30	18.7	18.1	19.6	26	-28
		NP1	278	0	278	19.7	0.32	19.5	18.4	20.2	26	-25
		Propyl gallate	232	0	232	21.9	0.39	21.8	20.6	23.0	24	-9
		Resveratrol	219	1	220	20.7	0.38	20.7	19.6	21.5	24	-14
	HK104	CTRL-DMSO	303	1	304	28.9	0.32	30.1	29.4	30.6	24	25
		NP1	399	0	399	24.0	0.39	24.6	23.6	25.3	28	-12
		Propyl gallate	358	4	362	29.7	0.35	30.4	29.7	31.0	35	-13
		Resveratrol	466	4	470	30.0	0.35	31.0	30.3	31.6	35	-11
	JU1348	CTRL-DMSO	319	1	320	19.2	0.28	18.5	17.6	19.3	25	-26
		NP1	278	0	278	19.7	0.35	19.4	18.9	20.2	28	-31
		Propyl gallate	333	0	333	20.1	0.30	19.7	19.2	20.3	25	-21
		Resveratrol	310	1	311	20.8	0.28	20.1	19.4	20.9	26	-23
