# Supplementary material for: Automated lifespan determination across Caenorhabditis strains and species reveals assay-specific effects of chemical interventions
Source: GeroScience. 2019 Dec 10;41(6):945–60. doi: 10.1007/s11357-019-00108-9 (PMC6925072; doi:10.1007/s11357-019-00108-9)
Supplement: Supplementary file 11 — Significance tests for compound interventions effects on longevity. Each effect is tested using both a general linear model of age at death and random effects Cox Proportional Hazard Model. Each compound is tested as a planned comparison against its appropriate carrier control. Variance components estimates for the randomized-block effects that were included in the overall model are presented in Online Resources 14–17 (PDF 155 kb) [file 11357_2019_108_MOESM11_ESM.pdf]

Online Resource 11 Significance tests for compound interventions effects on longevity. Each effect is tested using both a general linear model of age at death and random effects Cox Proportional Hazard Model. Each compound is tested as a planned comparison against its appropriate carrier control. Variance components estimates for the randomized-block effects that were included in the overall model are presented in Online Resources 14-17.

A. ***C. elegans*** **N2**

	General linear model				Random effects Cox Proportional Hazard			
Compound	Effect	Std err	*z*-value	p-value	Effect	Std err	*z*-value	*p*-value
NP1	3.74	0.33	11.39	<1E-04	-1.41	0.14	-10.21	<1E-04
Propyl gallate	1.32	0.36	3.65	0.0007	-0.56	0.15	-3.70	0.0006
Resveratrol	1.64	0.32	5.10	<1E-04	-0.77	0.13	-5.78	<1E-04
Thio T, unfiltered	-5.96	0.96	-6.23	<1E-09	2.79	0.23	12.01	<1E-10
Thio T, filtered	5.31	0.82	6.48	<1E-10	-1.18	0.19	-6.36	<1E-09
AKG, unfiltered	-2.14	0.57	-3.75	<0.001	1.23	0.26	4.80	<0.001
AKG, pH adjusted	-0.68	0.46	-1.50	0.2800	0.62	0.23	2.72	0.0163
AKG, unfilt, Phillips	-2.11	0.78	-2.69	0.0328	1.41	0.22	6.34	<0.001
AKG, filt, Phillips	1.02	0.74	1.38	0.4865	0.14	0.21	0.67	0.9009
AKG, pH adjusted, Phillips	-0.79	0.85	-0.93	0.7730	0.35	0.24	1.49	0.4184

B. ***C. elegans*** **MY16**

	General linear model				Random effects Cox Proportional Hazard			
Compound	Effect	Std err	*z*-value	*p*-value	Effect	Std err	*z*-value	*p*-value
NP1	1.69	0.78	2.17	0.0754	-0.45	0.24	-1.85	0.1543
Propyl gallate	1.83	0.76	2.41	0.0408	-0.86	0.24	-3.59	<0.001
Resveratrol	2.07	0.76	2.72	0.0169	-0.77	0.24	-3.26	0.0031
Thio T, unfiltered	1.03	1.86	0.56	0.8340	-0.21	0.32	-0.67	0.7665
Thio T, filtered	8.91	1.00	8.92	<0.001	-1.43	0.17	-8.54	<0.001
AKG, unfiltered	2.36	0.82	2.88	0.0102	-0.33	0.24	-1.40	0.3240
AKG, pH adjusted	0.19	0.64	0.30	0.9502	0.08	0.19	0.42	0.9050
AKG, unfilt, Phillips	1.04	0.70	1.49	0.4210	-0.01	0.16	-0.06	0.9999
AKG, filt, Phillips	0.27	0.54	0.51	0.9520	0.34	0.13	2.67	0.0348
AKG, pH adjusted, Phillips	-0.52	0.53	-0.98	0.7390	0.17	0.12	1.37	0.4944

C. *C. elegans* JU775

	General linear model				Random effects Cox Proportional Hazard			
Compound	Effect	Std err	*z*-value	*p*-value	Effect	Std err	*z*-value	*p*-value
NP1	4.85	0.50	9.70	<1E-07	-1.04	0.14	-7.59	<1E-05
Propyl gallate	2.84	0.51	5.58	<1E-07	-0.71	0.14	-5.08	<1E-05
Resveratrol	3.12	0.55	5.68	<1E-07	-0.94	0.15	-6.22	<1E-05
Thio T, unfiltered	-6.63	1.53	-4.32	0.0000	2.55	0.32	7.92	<1E-06
Thio T, filtered	10.16	1.23	8.24	<1E-05	-1.20	0.26	-4.68	<1E-05
AKG, unfiltered	1.05	0.95	1.11	0.4950	-0.01	0.22	-0.04	0.9990
AKG, pH adjusted	-1.34	0.83	-1.62	0.2250	0.34	0.19	1.85	0.1450
AKG, unfilt, Phillips	0.08	1.13	0.07	0.9999	0.15	0.22	0.68	0.8951
AKG, filt, Phillips	1.82	0.94	1.94	0.1951	0.00	0.19	0.01	1.0000
AKG, pH adjusted, Phillips	-3.46	0.96	-3.61	0.0021	0.69	0.22	3.21	0.0064

D. *C. briggsae* AF16

	General linear model				Random effects Cox Proportional Hazard			
Compound	Effect	Std err	*z*-value	*p*-value	Effect	Std err	*z*-value	*p*-value
NP1	0.49	0.88	0.56	0.9066	-0.15	0.16	-0.94	0.6828
Propyl gallate	1.94	0.88	2.21	0.0733	-0.48	0.16	-2.94	0.0093
Resveratrol	1.75	0.90	1.95	0.1324	-0.52	0.17	-3.08	0.0061
Thio T, unfiltered	-12.09	1.04	-11.62	<1E-04	5.33	0.34	15.72	<1E-05
Thio T, filtered	-1.04	1.22	-0.85	0.6590	-0.15	0.27	-0.58	0.8210
AKG, unfiltered	-3.79	0.78	-4.88	<1E-04	1.12	0.18	6.11	<1E-04
AKG, pH adjusted	-3.47	0.74	-4.70	<1E-04	0.96	0.17	5.65	<1E-04
AKG, unfilt, Phillips	-6.04	0.84	-7.15	<0.001	1.65	0.19	8.50	<0.001
AKG, filt, Phillips	1.78	0.80	2.23	0.1055	-0.29	0.18	-1.65	0.3276
AKG, pH adjusted, Phillips	-3.11	0.57	-5.43	<0.001	0.94	0.13	7.33	<0.001

E. *C. briggsae* HK104

	General linear model				Random effects Cox Proportional Hazard			
Compound	Effect	Std err	*z*-value	*p*-value	Effect	Std err	*z*-value	*p*-value
NP1	-4.04	0.90	-4.48	<0.001	0.52	0.20	2.58	0.0256
Propyl gallate	1.67	0.89	1.89	0.1404	-0.35	0.19	-1.80	0.1684
Resveratrol	2.10	0.86	2.44	0.0376	-0.56	0.19	-2.97	0.0081
Thio T, unfiltered	-14.71	2.12	-6.96	<0.001	3.64	0.40	9.15	<0.001
Thio T, filtered	3.81	1.46	2.61	0.0227	-0.65	0.26	-2.53	0.0283
AKG, unfiltered	-3.29	1.35	-2.44	0.0367	0.98	0.32	3.04	0.0061
AKG, pH adjusted	-7.16	1.26	-5.68	<0.001	1.62	0.31	5.21	<0.001
AKG, unfilt, Phillips	-5.85	1.26	-4.64	<0.001	1.67	0.29	5.68	<0.001
AKG, filt, Phillips	-4.01	1.64	-2.44	0.0633	0.51	0.37	1.37	0.4940
AKG, pH adjusted, Phillips	-5.76	1.26	-4.57	<0.001	1.10	0.29	3.78	<0.001

F. *C. briggsae* JU1348

	General linear model				Random effects Cox Proportional Hazard			
Compound	Effect	Std err	*z*-value	*p*-value	Effect	Std err	*z*-value	*p*-value
NP1	0.32	0.64	0.51	0.9274	-0.20	0.14	-1.39	0.3757
Propyl gallate	0.90	0.61	1.48	0.3234	-0.24	0.14	-1.73	0.0118
Resveratrol	1.60	0.62	2.60	0.0263	-0.39	0.14	-2.86	0.2064
Thio T, unfiltered	-11.98	1.61	-7.44	<0.001	6.16	0.35	17.65	<0.001
Thio T, filtered	2.61	1.19	2.20	0.0659	-0.35	0.13	-2.72	0.0155
AKG, unfiltered	0.69	1.59	0.43	0.8979	-0.26	0.34	-0.78	0.7006
AKG, pH adjusted	-4.52	1.17	-3.86	<0.001	1.10	0.26	4.24	<0.001
AKG, unfilt, Phillips	0.68	2.76	0.25	0.9940	-0.16	0.55	-0.29	0.9909
AKG, filt, Phillips	-6.94	2.21	-3.14	0.0082	1.28	0.45	2.83	0.0214
AKG, pH adjusted, Phillips	-5.77	1.57	-3.68	0.0011	1.25	0.32	3.94	<0.001
