# Supplementary material for: Automated lifespan determination across Caenorhabditis strains and species reveals assay-specific effects of chemical interventions
Source: GeroScience. 2019 Dec 10;41(6):945–60. doi: 10.1007/s11357-019-00108-9 (PMC6925072; doi:10.1007/s11357-019-00108-9)
Supplement: Supplementary file 12 — Significance tests for compound interventions effects on longevity. Each effect is tested using both a general linear model of age at death and random effects Cox Proportional Hazard Model. Each compound is tested as a planned comparison against its appropriate carrier control. Variance components estimates for the randomized-block effects that were included in the overall model are presented in Online Resources 5–17 (PDF 167 kb) [file 11357_2019_108_MOESM12_ESM.pdf]

Online Resource 12 Significance tests for compound interventions effects on longevity. Each effect is tested using both a general linear model of age at death and random effects Cox Proportional Hazard Model. Each compound is tested as a planned comparison against its appropriate carrier control. Variance components estimates for the randomized-block effects that were included in the overall model are presented in Online Resources 5-17.

A. *C. elegans* N2

	General linear model				Random effects Cox Proportional Hazard			
Compound	Effect	Std err	*z*-value	*p*-value	Effect	Std err	*z*-value	*p*-value
Thio T, filtered & unfiltered	-11.27	1.25	-8.98	<1E-10	3.97	0.30	13.33	<1E-10
AKG, unfiltered & pH adjusted	-1.45	0.73	-1.99	0.1070	0.61	0.36	1.67	0.2007
AKG, unfiltered & filtered	-3.13	1.08	-2.90	0.0180	1.27	0.30	4.19	<0.001
AKG, pH adjusted & filtered	-1.81	1.13	-1.60	0.3530	0.21	0.31	0.68	0.8950
AKG, pH adjusted & unfiltered	1.32	1.16	1.14	0.6449	-1.06	0.32	-3.28	0.0053

B. *C. elegans* MY16

	General linear model				Random effects Cox Proportional Hazard			
Compound	Effect	Std err	*z*-value	*p*-value	Effect	Std err	*z*-value	*p*-value
Thio T, filtered & unfiltered	-7.88	2.11	-3.74	<0.001	1.22	0.36	3.39	0.0017
AKG, unfiltered & pH adjusted	2.17	1.04	2.09	0.0858	-0.41	0.30	-1.35	0.3500
AKG, unfiltered & filtered	0.76	0.88	0.87	0.8070	-0.35	0.20	-1.74	0.2844
AKG, pH adjusted & filtered	-0.79	0.75	-1.05	0.6960	0.18	0.17	-1.01	0.7244
AKG, pH adjusted & unfiltered	-1.56	0.88	-1.78	0.2630	0.17	0.20	0.88	0.8000

C. *C. elegans* JU775

	General linear model				Random effects Cox Proportional Hazard			
Compound	Effect	Std err	*z*-value	*p*-value	Effect	Std err	*z*-value	*p*-value
Thio T, filtered & unfiltered	-16.78	1.95	-8.61	<1E-05	3.74	0.41	9.11	<1E-06
AKG, unfiltered & pH adjusted	2.40	1.26	1.90	0.1310	-0.35	0.29	-1.21	0.4290
AKG, unfiltered & filtered	-1.74	1.47	-1.18	0.6140	0.14	0.28	0.51	0.9528
AKG, pH adjusted & filtered	-5.28	1.34	-3.94	<0.001	0.69	0.28	2.43	0.0651

AKG, pH adjusted & unfiltered	-3.54	1.48	-2.39	0.0721	0.55	0.30	1.80	0.2535

D. *C. briggsae* AF16

	General linear model				Random effects Cox Proportional Hazard			
Compound	Effect	Std err	*z*-value	*p*-value	Effect	Std err	*z*-value	*p*-value
Thio T, filtered & unfiltered	-11.05	1.60	-6.92	<1E-04	5.48	0.43	12.83	<1E-05
AKG, unfiltered & pH adjusted	-0.31	1.07	-0.29	0.9510	0.16	0.25	0.63	0.7940
AKG, unfiltered & filtered	-7.82	1.16	-6.72	<0.001	1.94	0.26	7.40	<0.001
AKG, pH adjusted & filtered	-4.89	0.98	-4.97	<0.001	1.23	0.22	5.64	<0.001
AKG, pH adjusted & unfiltered	2.92	1.02	2.86	0.0195	-0.71	0.23	-3.12	0.0086

E. *C. briggsae* HK104

	General linear model				Random effects Cox Proportional Hazard			
Compound	Effect	Std err	z-value	p-value	Effect	Std err	z-value	p-value
Thio T, filtered & unfiltered	-18.52	2.55	-7.25	<0.001	4.29	0.47	9.12	<0.001
AKG, unfiltered & pH adjusted	3.87	1.83	2.12	0.0815	-0.64	0.45	-1.42	0.3154
AKG, unfiltered & filtered	-1.84	2.09	-0.88	0.7988	1.16	0.48	2.44	0.0638
AKG, pH adjusted & filtered	-1.75	2.07	-0.85	0.8170	0.60	0.47	1.27	0.5568
AKG, pH adjusted & unfiltered	0.09	1.77	0.05	1.0000	-0.57	0.41	-1.38	0.4864

F. *C. briggsae* JU1348

	General linear model				Random effects Cox Proportional Hazard			
Compound	Effect	Std err	z-value	p-value	Effect	Std err	z-value	p-value
Thio T, filtered & unfiltered	-14.60	1.99	-7.34	<0.001	6.50	0.37	17.47	<0.001
AKG, unfiltered & pH adjusted	5.21	1.97	2.65	0.0201	-1.36	0.43	-3.20	0.0038
AKG, unfiltered & filtered	7.62	3.54	2.16	0.1221	-1.43	0.71	-2.02	0.1623
AKG, pH adjusted & filtered	1.17	2.71	0.43	0.9695	-0.03	0.55	-0.05	1.0000
AKG, pH adjusted & unfiltered	-6.45	3.17	-2.03	0.1592	1.41	0.63	2.23	0.1049
