# Supplementary material for: Automated lifespan determination across Caenorhabditis strains and species reveals assay-specific effects of chemical interventions
Source: GeroScience. 2019 Dec 10;41(6):945–60. doi: 10.1007/s11357-019-00108-9 (PMC6925072; doi:10.1007/s11357-019-00108-9)
Supplement: Supplementary file 13 — Summary of ALM lifespan data under compound treatment (AKG and ThT) conditions, and comparison to median lifespan from comparable manual assays (PDF 128 kb) [file 11357_2019_108_MOESM13_ESM.pdf]

Online Resource 13 Summary of ALM lifespan data under compound treatment (AKG and ThT) conditions, and comparison to median lifespan from comparable manual assays

				ALM								Manual	
**Species**	**Strain**	**Comp**	**Condition**	**Number of deaths**	**Number censored**	**Total obs.**	**Mean lifespan**	**SEM**	**Median lifespan**	**Lower 95% CI**	**Upper 95% CI**	**Median lifespan**	**% diff med LS from manual**
C. elegans	JU775	AKG	filter	184	0	184	24.2	0.45	24.2	22.9	25.3		
			nofilter	297	0	297	18.2	0.28	17.2	16.7	18.1	25	-31
			pH adjusted	550	0	550	17.3	0.20	16.9	16.5	17.2		
		CTRL -H2O	filter	554	5	559	19.4	0.35	16.0	15.6	16.4		
			nofilter	447	0	447	16.8	0.23	15.9	15.4	16.6	21	-24
			pH adjusted	514	0	514	18.8	0.29	17.0	16.5	17.4		
		ThT	filter	250	0	250	29.0	0.46	29.1	28.4	29.8		
			nofilter	115	0	115	11.5	0.33	10.3	9.9	11.4	28	-63
	MY16	AKG	filter	134	0	134	18.3	0.24	18.1	17.4	18.5		
			nofilter	182	0	182	17.7	0.27	17.0	16.7	17.8	25	-32
			pH adjusted	492	0	492	17.7	0.21	17.3	16.7	17.8		
		CTRL -H2O	filter	384	2	386	17.6	0.30	15.9	14.8	16.8		
			nofilter	307	0	307	15.2	0.23	14.6	14.0	15.4	16	-8
			pH adjusted	454	0	454	17.2	0.28	16.5	16.0	17.0		
		ThT	filter	181	2	183	27.0	0.53	28.6	27.1	29.5		
			nofilter	43	0	43	17.7	0.69	18.7	17.3	19.4	28	-33
	N2	AKG	filter	181	0	181	19.8	0.24	19.3	18.7	20.1		
			nofilter	327	1	328	14.1	0.11	14.0	13.9	14.3	23	-39
			pH adjusted	560	0	560	16.8	0.14	16.7	16.3	17.1		
		CTRL -H2O	filter	495	1	496	18.0	0.23	18.0	17.1	18.5		
			nofilter	513	0	513	15.9	0.13	16.5	16.2	16.8	18	-8
			pH adjusted	660	0	660	17.0	0.13	17.5	17.2	17.8		
		ThT	filter	392	3	395	23.9	0.36	24.9	23.9	25.6		
			nofilter	148	0	148	9.8	0.16	9.4	8.9	9.7	23	-59
C. briggsae	AF16	AKG	filter	75	0	75	26.5	0.62	27.1	24.4	28.6		
			nofilter	163	0	163	17.1	0.32	16.6	16.2	17.5	28	-41
			pH adjusted	325	0	325	17.0	0.21	16.7	16.2	17.1		
		CTRL -H2O	filter	213	1	214	23.0	0.43	23.2	22.3	24.5		
			nofilter	325	2	327	20.8	0.25	21.1	20.6	21.7	26	-19
			pH adjusted	392	0	392	19.9	0.28	19.4	18.6	20.3		
		ThT	filter	70	0	70	21.3	1.21	19.6	14.4	24.1		
			nofilter	109	0	109	9.1	0.06	9.1	9.0	9.2	32	-72
	HK104	AKG	filter	64	0	64	34.3	1.13	36.8	33.8	39.3		
			nofilter	318	5	323	23.5	0.39	22.5	21.5	22.9	35	-36
			pH adjusted	406	0	406	23.6	0.31	24.3	23.1	25.0		
		CTRL -H2O	filter	282	1	283	35.1	0.60	36.8	35.2	37.7		
			nofilter	488	5	493	26.5	0.30	26.5	25.8	27.2	38	-30
			pH adjusted	553	0	553	30.6	0.28	31.5	30.9	32.1		
		ThT	filter	146	2	148	40.4	1.13	44.1	42.0	45.8		
			nofilter	93	1	94	13.6	0.47	12.0	11.6	12.3	42	-71
	JU1348	AKG	filter	57	0	57	20.4	0.59	19.1	18.7	20.0		
			nofilter	105	0	105	20.9	0.46	20.4	19.6	21.2	25	-18
			pH adjusted	432	0	432	16.4	0.22	15.7	15.2	16.2		
		CTRL -H2O	filter	208	0	208	24.1	0.57	23.0	21.6	24.1		
			nofilter	279	0	279	19.2	0.36	18.0	17.3	18.4	26	-31
			pH adjusted	337	0	337	21.1	0.30	20.4	19.8	21.5		
		ThT	filter	172	0	172	26.2	0.46	25.7	24.6	26.5		
			nofilter	72	0	72	10.0	0.14	9.8	9.2	10.6	28	-65
