# Supplementary material for: Automated lifespan determination across Caenorhabditis strains and species reveals assay-specific effects of chemical interventions
Source: GeroScience. 2019 Dec 10;41(6):945–60. doi: 10.1007/s11357-019-00108-9 (PMC6925072; doi:10.1007/s11357-019-00108-9)
Supplement: Supplementary file 14 — Variance components estimates for longevity for the NP1, PG, and RVL compound experiments, analyzed separately for each strain. Values are from a hierarchical randomized block design estimated either via a restricted maximum likelihood general linear model using the lme4 package (v. 1.1-21) or via a random effects Cox Proportional Hazards model as implemented by the coxme package (v. 2.2-10) in R (Therneau 2012) (PDF 142 kb) [file 11357_2019_108_MOESM14_ESM.pdf]

Online Resource 14 Variance components estimates for longevity for the NP1, PG, and RVL compound experiments, analyzed separately for each strain. Values are from a hierarchical randomized block design estimated either via a restricted maximum likelihood general linear model using the *lme4* package (v. 1.1-21) or via a random effects Cox Proportional Hazards model as implemented by the *coxme* package (v. 2.2-10) in R (Therneau 2012).

A. ***C. elegans*** **N2** (*n* = 1,806)

	General Linear Model				Cox Prop Hazard
**Source**	**Var Comp**	**Lower 95% CI**	**Upper 95% CI**	**Percent Total**	**Var Comp**
Lab	1.09	0.00	8.32	9.9	0.08
Scanner[Lab]	0.00	0.00	3.10	0.0	0.01
Trial[Lab,Scn]	1.55	0.18	3.94	14.1	0.39
Plate-T[Lab,Scn,Trial]	0.33	0.08	0.67	3.0	0.06
Residual	8.01	7.51	8.57	72.9	
Total	10.99			100.0	

B. ***C. elegans*** **MY16** (*n* = 1,539)

	General Linear Model				Cox Prop Hazard
**Source**	**Var Comp**	**Lower 95% CI**	**Upper 95% CI**	**Percent Total**	**Var Comp**
Lab	0.15	0.00	4.46	1.0	0.00
Scanner[Lab]	0.00	0.00	2.78	0.0	0.01
Trial[Lab,Scn]	2.15	0.69	5.53	13.7	0.12
Plate-T[Lab,Scn,Trial]	2.43	1.28	3.89	15.6	0.26
Residual	10.92	10.17	11.74	69.8	
Total	15.65			100.0	

C. ***C. elegans*** **JU775** (*n* = 1,695)

	General Linear Model				Cox Prop Hazard
**Source**	**Var Comp**	**Lower 95% CI**	**Upper 95% CI**	**Percent Total**	**Var Comp**
Lab	0.00	0.00	1.78	0.0	0.02
Scanner[Lab]	0.00	0.00	1.50	0.0	0.00
Trial[Lab,Scn]	1.95	0.77	4.45	8.7	0.11
Plate-T[Lab,Scn,Trial]	0.72	0.15	1.45	3.2	0.07
Residual	19.79	18.50	21.20	88.1	
Total	22.46			100.0	

D. ***C. briggsae*** **AF16** (*n* = 1,029)

Source	General Linear Model				Cox Prop Hazard
	Var Comp	Lower 95% CI	Upper 95% CI	Percent Total	Var Comp
Lab	0.00	0.00	2.98	0.0	0.00
Scanner[Lab]	0.00	0.00	2.99	0.0	0.00
Trial[Lab,Scn]	2.81	0.63	7.64	9.0	0.17
Plate-T[Lab,Scn,Trial]	3.12	1.29	5.39	10.0	0.10
Residual	25.13	23.03	27.49	80.9	
Total	31.06			100.0	

E. ***C. briggsae*** **HK104** (*n* = 1,535)

Source	General Linear Model				Cox Prop Hazard
	Var Comp	Lower 95% CI	Upper 95% CI	Percent Total	Var Comp
Lab	17.35	2.99	103.42	31.4	0.47
Scanner[Lab]	0.79	0.00	4.22	1.4	0.07
Trial[Lab,Scn]	0.00	0.00	3.19	0.0	0.00
Plate-T[Lab,Scn,Trial]	2.57	1.10	4.75	4.7	0.14
Residual	34.54	32.18	37.14	62.5	
Total	55.24			100.0	

F. ***C. briggsae*** **JU1348** (*n* = 1,242)

Source	General Linear Model				Cox Prop Hazard
	Var Comp	Lower 95% CI	Upper 95% CI	Percent Total	Var Comp
Lab	4.55	0.00	31.24	14.8	0.19
Scanner[Lab]	2.01	0.64	7.02	6.5	0.14
Trial[Lab,Scn]	0.00	0.00	4.24	0.0	0.00
Plate-T[Lab,Scn,Trial]	1.29	0.42	2.39	4.2	0.07
Residual	22.85	21.11	24.77	74.4	
Total	30.70			100.0	

Reference List

Therneau, T. (2012) coxme: Mixed Effects Cox Models. R package version 2.2-3. Available at: http://CRAN.R-project.org/package=coxme
