# Supplementary material for: Automated lifespan determination across Caenorhabditis strains and species reveals assay-specific effects of chemical interventions
Source: GeroScience. 2019 Dec 10;41(6):945–60. doi: 10.1007/s11357-019-00108-9 (PMC6925072; doi:10.1007/s11357-019-00108-9)
Supplement: Supplementary file 15 — Variance components estimates for longevity for the Thioflavin T filtered and unfiltered compound experiments, analyzed separately for each strain. Values are from a hierarchical randomized block design estimated either via a restricted maximum likelihood general linear model using the lme4 package (v. 1.1-21) or via a random effects Cox Proportional Hazards model as implemented by the coxme package (v. 2.2-10) in R (Therneau 2012) (PDF 170 kb) [file 11357_2019_108_MOESM15_ESM.pdf]

Online Resource 15 Variance components estimates for longevity for the Thioflavin T filtered and unfiltered compound experiments, analyzed separately for each strain. Values are from a hierarchical randomized block design estimated either via a restricted maximum likelihood general linear model using the *lme4* package (v. 1.1-21) or via a random effects Cox Proportional Hazards model as implemented by the *coxme* package (v. 2.2-10) in R (Therneau 2012).

A. *C. elegans* N2 (*n* = 1,552)

Source	General Linear Model				Cox Prop Hazard
	Var Comp	Lower 95% CI	Upper 95% CI	Percent Total	Var Comp
Lab	0.33	0.00	3.14	1.29	0.00
Scanner[Lab]	0.00	0.00	2.12	0.00	0.02
Trial[Lab,Scn]	0.18	0.00	2.00	0.72	0.02
Plate-T[Lab,Scn,Trial]	2.90	1.08	4.93	11.54	0.14
Residual	21.76	20.27	23.39	86.45	
Total	25.17			100.0	

B. *C. elegans* MY16 (*n* = 919)

Source	General Linear Model				Cox Prop Hazard
	Var Comp	Lower 95% CI	Upper 95% CI	Percent Total	Var Comp
Lab	0.00	0.00	4.59	0.00	0.00
Scanner[Lab]	2.01	0.00	12.46	5.99	0.00
Trial[Lab,Scn]	4.33	1.36	13.08	12.94	0.22
Plate-T[Lab,Scn,Trial]	2.81	0.49	6.39	8.41	0.05
Residual	24.31	22.18	26.73	72.66	
Total	33.45			100.0	

C. *C. elegans* JU775 (*n* = 1,371)

Source	General Linear Model				Cox Prop Hazard
	Var Comp	Lower 95% CI	Upper 95% CI	Percent Total	Var Comp
Lab	0.71	0.00	8.44	1.57	0.00
Scanner[Lab]	3.23	0.00	9.58	7.12	0.12
Trial[Lab,Scn]	0.00	0.00	6.19	0.00	0.00
Plate-T[Lab,Scn,Trial]	6.04	2.57	10.49	13.32	0.29
Residual	35.34	32.77	38.16	77.99	
Total	45.31			100.0	

D. ***C. briggsae*** **AF16** (*n* = 720)

Source	General Linear Model				Cox Prop Hazard
	Var Comp	Lower 95% CI	Upper 95% CI	Percent Total	Var Comp
Lab	2.68	0.00	21.07	7.64	0.31
Scanner[Lab]	0.00	0.00	3.84	0.00	0.00
Trial[Lab,Scn]	5.70	0.00	13.58	16.23	0.47
Plate-T[Lab,Scn,Trial]	1.91	0.00	17.51	5.43	0.08
Residual	24.83	22.37	27.68	70.70	
Total	35.12			100.0	

E. ***C. briggsae*** **HK104** (*n* = 1,018)

Source	General Linear Model				Cox Prop Hazard
	Var Comp	Lower 95% CI	Upper 95% CI	Percent Total	Var Comp
Lab	41.15	0.00	13.08	35.94	0.85
Scanner[Lab]	16.43	4.45	45.17	14.35	1.07
Trial[Lab,Scn]	0.00	0.00	10.94	0.00	0.00
Plate-T[Lab,Scn,Trial]	7.04	2.75	13.08	6.15	0.23
Residual	49.86	45.69	54.55	43.55	
Total	114.49			100.0	

F. ***C. briggsae*** **JU1348** (*n* = 731)

Source	General Linear Model				Cox Prop Hazard
	Var Comp	Lower 95% CI	Upper 95% CI	Percent Total	Var Comp
Lab	3.35	0.00	28.55	6.87	0.03
Scanner[Lab]	8.29	0.00	24.56	17.01	0.93
Trial[Lab,Scn]	0.00	0.00	8.70	0.00	0.00
Plate-T[Lab,Scn,Trial]	3.23	0.46	7.24	6.62	0.01
Residual	33.88	30.58	37.72	69.50	
Total	48.74			100.0	

Reference List

Therneau, T. (2012) coxme: Mixed Effects Cox Models. R package version 2.2-3. Available at: http://CRAN.R-project.org/package=coxme
