# Supplementary material for: Automated lifespan determination across Caenorhabditis strains and species reveals assay-specific effects of chemical interventions
Source: GeroScience. 2019 Dec 10;41(6):945–60. doi: 10.1007/s11357-019-00108-9 (PMC6925072; doi:10.1007/s11357-019-00108-9)
Supplement: Supplementary file 16 — Variance components estimates for longevity for the α-ketoglutarate pH adjusted and unfiltered compound experiments, analyzed separately for each strain. Values are from a hierarchical randomized block design estimated either via a restricted maximum likelihood general linear model using the lme4 package (v. 1.1-21) or via a random effects Cox Proportional Hazards model as implemented by the coxme package (v. 2.2-10) in R (Therneau 2012) (PDF 184 kb) [file 11357_2019_108_MOESM16_ESM.pdf]

Online Resource 16 Variance components estimates for longevity for the α-ketoglutarate pH adjusted and unfiltered compound experiments, analyzed separately for each strain. Values are from a hierarchical randomized block design estimated either via a restricted maximum likelihood general linear model using the *lme4* package (v. 1.1-21) or via a random effects Cox Proportional Hazards model as implemented by the *coxme* package (v. 2.2-10) in R (Therneau 2012).

A. *C. elegans* N2 (*n* = 2,061)

	General Linear Model				Cox Prop Hazard
Source	Var Comp	Lower 95% CI	Upper 95% CI	Percent Total	Var Comp
Lab	0.75	0.00	5.80	7.35	0.20
Scanner[Lab]	0.00	0.00	2.65	0.00	0.00
Trial[Lab,Scn]	2.02	0.01	4.32	19.79	0.20
Plate-T[Lab,Scn,Trial]	1.31	0.63	2.27	12.86	0.35
Residual	6.13	5.77	6.53	60.00	
Total	10.22			100.0	

B. *C. elegans* MY16 (*n* = 1,435)

	General Linear Model				Cox Prop Hazard
Source	Var Comp	Lower 95% CI	Upper 95% CI	Percent Total	Var Comp
Lab	0.00	0.00	2.46	0.00	0.00
Scanner[Lab]	2.67	0.00	7.61	11.17	0.23
Trial[Lab,Scn]	1.07	0.00	10.87	4.47	0.01
Plate-T[Lab,Scn,Trial]	1.63	0.58	3.43	6.84	0.16
Residual	18.51	17.21	19.96	77.53	
Total	23.87			100.0	

C. *C. elegans* JU775 (*n* = 1,808)

	General Linear Model				Cox Prop Hazard
Source	Var Comp	Lower 95% CI	Upper 95% CI	Percent Total	Var Comp
Lab	0.43				0.04
Scanner[Lab]	0.00				0.22
Trial[Lab,Scn]	4.38				0.04
Plate-T[Lab,Scn,Trial]	3.70				0.18
Residual	19.25				
Total	27.76				

D. *C. briggsae* AF16 (*n* = 1,207)

Source	General Linear Model				Cox Prop Hazard
	Var Comp	Lower 95% CI	Upper 95% CI	Percent Total	Var Comp
Lab	0.29	0.00	3.98	1.30	0.00
Scanner[Lab]	2.04	0.00	5.77	9.09	0.12
Trial[Lab,Scn]	0.02	0.00	8.08	0.08	0.00
Plate-T[Lab,Scn,Trial]	2.33	0.47	4.28	10.39	0.12
Residual	17.73	16.37	19.28	79.15	
Total	22.40			100.0	

E. *C. briggsae* HK104 (*n* = 1,775)

Source	General Linear Model				Cox Prop Hazard
	Var Comp	Lower 95% CI	Upper 95% CI	Percent Total	Var Comp
Lab	0.01	0.00	14.15	0.02	0.01
Scanner[Lab]	7.50	1.90	13.59	15.84	0.31
Trial[Lab,Scn]	0.00	0.00	18.63	0.00	0.01
Plate-T[Lab,Scn,Trial]	8.65	4.63	11.38	18.27	0.53
Residual	31.19	29.19	33.37	65.87	
Total	47.34			100.0	

F. *C. briggsae* JU1348 (*n* = 1,153)

Source	General Linear Model				Cox Prop Hazard
	Var Comp	Lower 95% CI	Upper 95% CI	Percent Total	Var Comp
Lab	0.79	0.00	7.19	2.67	0.00
Scanner[Lab]	0.00	0.00	4.93	0.00	0.00
Trial[Lab,Scn]	0.00	0.00	4.68	0.00	0.00
Plate-T[Lab,Scn,Trial]	7.37	3.29	11.57	24.94	0.36
Residual	21.39	19.71	23.28	72.39	
Total	29.55			100.0	

Reference List

Therneau, T. (2012) coxme: Mixed Effects Cox Models. R package version 2.2-3. Available at: http://CRAN.R-project.org/package=coxme
