# Supplementary material for: Automated lifespan determination across Caenorhabditis strains and species reveals assay-specific effects of chemical interventions
Source: GeroScience. 2019 Dec 10;41(6):945–60. doi: 10.1007/s11357-019-00108-9 (PMC6925072; doi:10.1007/s11357-019-00108-9)
Supplement: Supplementary file 17 — Variance components estimates for longevity for the α-ketoglutarate pH adjusted, filtered, and unfiltered compound experiments from the Phillips laboratory, analyzed separately for each strain. Values are from a hierarchical randomized block design estimated either via a restricted maximum likelihood general linear model using the lme4 package (v. 1.1-21) or via a random effects Cox Proportional Hazards model as implemented by the coxme package (v. 2.2-10) in R (Therneau 2012) (PDF 182 kb) [file 11357_2019_108_MOESM17_ESM.pdf]

Online Resource 17 Variance components estimates for longevity for the α-ketoglutarate pH adjusted, filtered, and unfiltered compound experiments from the Phillips laboratory, analyzed separately for each strain. Values are from a hierarchical randomized block design estimated either via a restricted maximum likelihood general linear model using the *lme4* package (v. 1.1-21) or via a random effects Cox Proportional Hazards model as implemented by the *coxme* package (v. 2.2-10) in R (Therneau 2012).

A. ***C. elegans*** **N2** (*n* = 1,436)

	General Linear Model				Cox Prop Hazard
**Source**	**Var Comp**	**Lower 95% CI**	**Upper 95% CI**	**Percent Total**	**Var Comp**
Scanner	0.00	0.00	0.42	0.00	0.00
Trial*Scanner	0.00	0.00	0.41	0.00	0.00
Plate-T[Scanner,Trial]	1.54	0.71	2.21	13.68	0.11
Residual	9.69	9.01	10.45	86.32	
Total	11.23			100.0	

B. ***C. elegans*** **MY16** (*n* = 856)

	General Linear Model				Cox Prop Hazard
**Source**	**Var Comp**	**Lower 95% CI**	**Upper 95% CI**	**Percent Total**	**Var Comp**
Scanner[Lab]	0.49	0.00	2.33	2.30	0.00
Trial[Lab,Scn]	0.92	0.00	2.73	4.33	0.07
Plate-T[Lab,Scn,Trial]	0.00	0.00	0.69	0.00	0.00
Residual	19.78	17.95	21.75	93.37	
Total	21.19			100.0	

C. ***C. elegans*** **JU775** (*n* = 1,372)

	General Linear Model				Cox Prop Hazard
**Source**	**Var Comp**	**Lower 95% CI**	**Upper 95% CI**	**Percent Total**	**Var Comp**
Scanner[Lab]	0.00	0.00	3.47	0.00	0.00
Trial[Lab,Scn]	1.97	0.00	4.65	4.93	0.00
Plate-T[Lab,Scn,Trial]	1.53	0.03	3.85	3.82	0.09
Residual	36.54	33.91	39.47	91.26	
Total	40.04			100.0	

D. *C. briggsae* AF16 (*n* = 744)

Source	General Linear Model				Cox Prop Hazard
	Var Comp	Lower 95% CI	Upper 95% CI	Percent Total	Var Comp
Scanner[Lab]	0.00	0.00	3.35	0.00	0.00
Trial[Lab,Scn]	3.93	0.81	6.98	15.66	0.16
Plate-T[Lab,Scn,Trial]	0.00	0.00	0.78	0.00	0.00
Residual	21.17	19.06	23.43	84.34	
Total	25.10			100.0	

E. *C. briggsae* HK104 (*n* = 1,179)

Source	General Linear Model				Cox Prop Hazard
	Var Comp	Lower 95% CI	Upper 95% CI	Percent Total	Var Comp
Scanner[Lab]	0.87	1.18	6.26	2.08	0.08
Trial[Lab,Scn]	0.01	0.00	4.35	0.01	0.01
Plate-T[Lab,Scn,Trial]	3.59	0.00	3.65	8.63	0.21
Residual	37.17	34.28	40.38	89.28	
Total	41.63			100.0	

F. *C. briggsae* JU1348 (*n* = 602)

Source	General Linear Model				Cox Prop Hazard
	Var Comp	Lower 95% CI	Upper 95% CI	Percent Total	Var Comp
Scanner[Lab]	0.00	0.00	3.46	0.00	0.00
Trial[Lab,Scn]	0.00	0.00	4.32	0.00	0.00
Plate-T[Lab,Scn,Trial]	5.13	1.26	7.80	13.17	0.22
Residual	33.80	30.42	37.71	86.83	
Total	38.93			100.0	

Reference List

Therneau, T. (2012) coxme: Mixed Effects Cox Models. R package version 2.2-3. Available at: http://CRAN.R-project.org/package=coxme
