# Supplementary material for: Automated lifespan determination across Caenorhabditis strains and species reveals assay-specific effects of chemical interventions
Source: GeroScience. 2019 Dec 10;41(6):945–60. doi: 10.1007/s11357-019-00108-9 (PMC6925072; doi:10.1007/s11357-019-00108-9)
Supplement: Supplementary file 18 — Thioflavin T is toxic under the intense illumination during automated lifespan analysis. Survivorship curves generated using automated lifespan analysis under control (black lines) or thioflavin T (mustard lines) under normal illumination (solid lines) or filtered (dash-dot lines) Vertical dotted line shows day plates introduced to the scanners and the start of intense illumination. Automated analysis resulted in shortened lifespan under thioflavin T exposure, while filtering the ALM light restored the lifespan extension of thioflavin T exposure (PDF 294 kb) [file 11357_2019_108_MOESM18_ESM.pdf]

Online Resource 18 Thioflavin T is toxic under the intense illumination during automated lifespan analysis

Survivorship curves generated using automated lifespan analysis under control (black lines) or thioflavin T (mustard lines) under normal illumination (solid lines) or filtered (dash-dot lines) Vertical dotted line shows day plates introduced to the scanners and the start of intense illumination. Automated analysis resulted in shortened lifespan under thioflavin T exposure, while filtering the ALM light restored the lifespan extension of thioflavin T exposure.

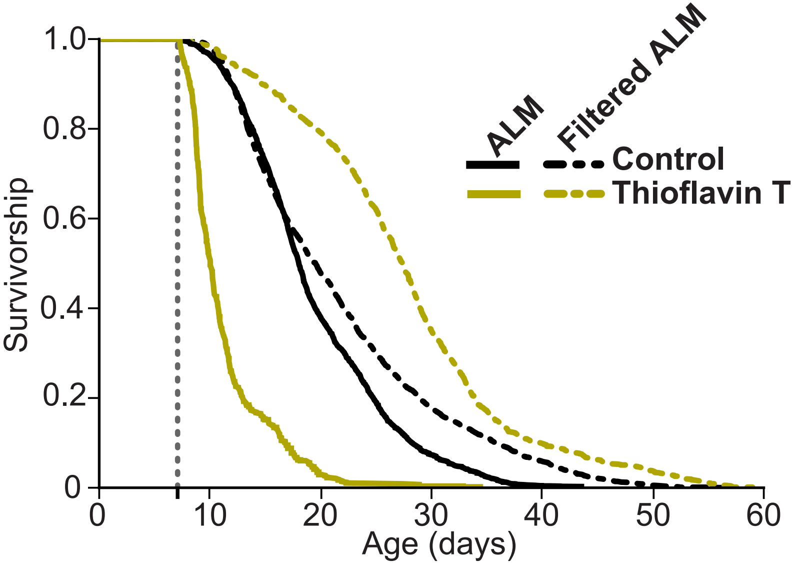

ALM
Filtered ALM
Control
Thioflavin T
Survivorship
1.0
0.8
0.6
0.4
0.2
0
0
10
20
30
40
50
60
Age (days)
