# Supplementary material for: Automated lifespan determination across Caenorhabditis strains and species reveals assay-specific effects of chemical interventions
Source: GeroScience. 2019 Dec 10;41(6):945–60. doi: 10.1007/s11357-019-00108-9 (PMC6925072; doi:10.1007/s11357-019-00108-9)
Supplement: Supplementary file 19 — Alpha ketoglutarate lifespan effects are not due to pH differences in automated lifespan analysis. The percent change in median lifespan from control for animals grown under adult exposure to α-ketoglutarate for three C. elegans (N2, JU775, and MY16) and C. briggsae (AF16, JU1348, and HK104) strains. Each point represents the change in median lifespan for an individual plate trial. Replicates were generated at three CITP sites (Blue-Buck Institute, Green-Oregon and Red- Rutgers). Lifespans were measured by standard manual lifespan analysis (open circles), ALM analysis (closed circles), or ALM analysis in which the α-ketoglutarate stock solutions had been adjusted to pH 6 (closed squares) prior to plate treatment (see materials and methods). Asterisks represent p values (****p<0.0001, *** p<0.001, ** p<0.01 and * p<0.05) from the CPH model when comparing the lifespans under compound exposure versus the lifespans exposed to the vehicle control (PDF 329 kb) [file 11357_2019_108_MOESM19_ESM.pdf]

Online Resource 19 Alpha ketoglutarate lifespan effects are not due to pH differences in automated lifespan analysis

The percent change in median lifespan from control for animals grown under adult exposure to α-ketoglutarate for three *C. elegans* (N2, JU775, and MY16) and *C. briggsae* (AF16, JU1348, and HK104) strains. Each point represents the change in median lifespan for an individual plate trial. Replicates were generated at three CITP sites (Blue-Buck Institute, Green-Oregon and Red- Rutgers). Lifespans were measured by standard manual lifespan analysis (open circles), ALM analysis (closed circles), or ALM analysis in which the α-ketoglutarate stock solutions had been adjusted to pH 6 (closed squares) prior to plate treatment (see materials and methods). Asterisks represent *p*-values (****$p<0.0001$, *** $p<0.001$, ** $p<0.01$ and * $p<0.05$) from the CPH model when comparing the lifespans under compound exposure versus the lifespans exposed to the vehicle control.

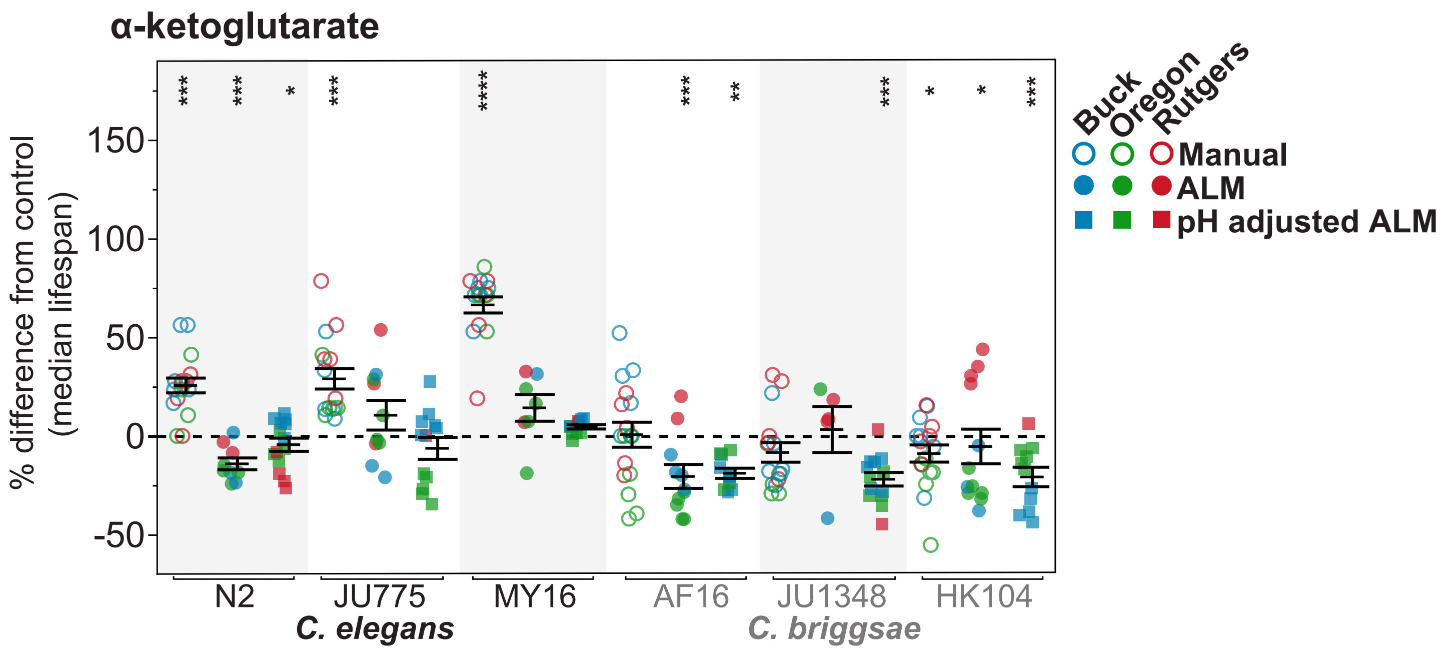

α-ketoglutarate
% difference from control
(median lifespan)
150
100
50
0
-50
***
***
*
***
****
***
**
***
*
*
***
Buck
Oregon
Rutgers
Manual
ALM
pH adjusted ALM
N2
JU775
MY16
AF16
JU1348
HK104
C. elegans
C. briggsae
