# Supplementary material for: Automated lifespan determination across Caenorhabditis strains and species reveals assay-specific effects of chemical interventions
Source: GeroScience. 2019 Dec 10;41(6):945–60. doi: 10.1007/s11357-019-00108-9 (PMC6925072; doi:10.1007/s11357-019-00108-9)
Supplement: Supplementary file 20 — Manual vs. ALM: approximate average return rates per plate (observed mean number of deaths versus expected number of animals at experiment start). The expected number of animals per plate for manual assays was 37.5, and for ALM assays was 50 (PDF 150 kb) [file 11357_2019_108_MOESM20_ESM.pdf]

Online Resource 20 Manual vs. ALM: approximate average return rates per plate (observed mean number of deaths versus expected number of animals at experiment start). The expected number of animals per plate for manual assays was 37.5, and for ALM assays was 50.

		Manual		ALM	
	**Strain**	**Observed deaths**	**% expected observed**	**Observed deaths**	**% expected observed**
C. elegans	N2	31	83	38	76
	N2 PD1073	-	-	33	66
	CB4856	31	83	30	60
	ED3040	33	88	37	74
	JU775	32	85	34	68
	JU1088	33	88	33	66
	JU1652	32	85	32	64
	MY16	31	83	30	60
	QX1211	31	83	26	52
C. briggsae	AF16	25	67	20	40
	ED3092	25	67	29	58
	HK104	25	67	29	58
	JU726	29	77	24	48
	JU1264	24	64	29	58
	JU1348	24	64	24	48
	NIC20	25	67	29	58
	QR25	25	67	32	64
C. tropicalis	JU1373	30	80	33	66
	JU1630	31	83	20	40
	NIC58	25	67	23	46
	NIC122	23	61	27	54
	QG131	25	67	27	54
	QG834	29	77	29	58
